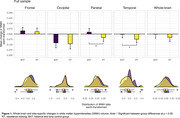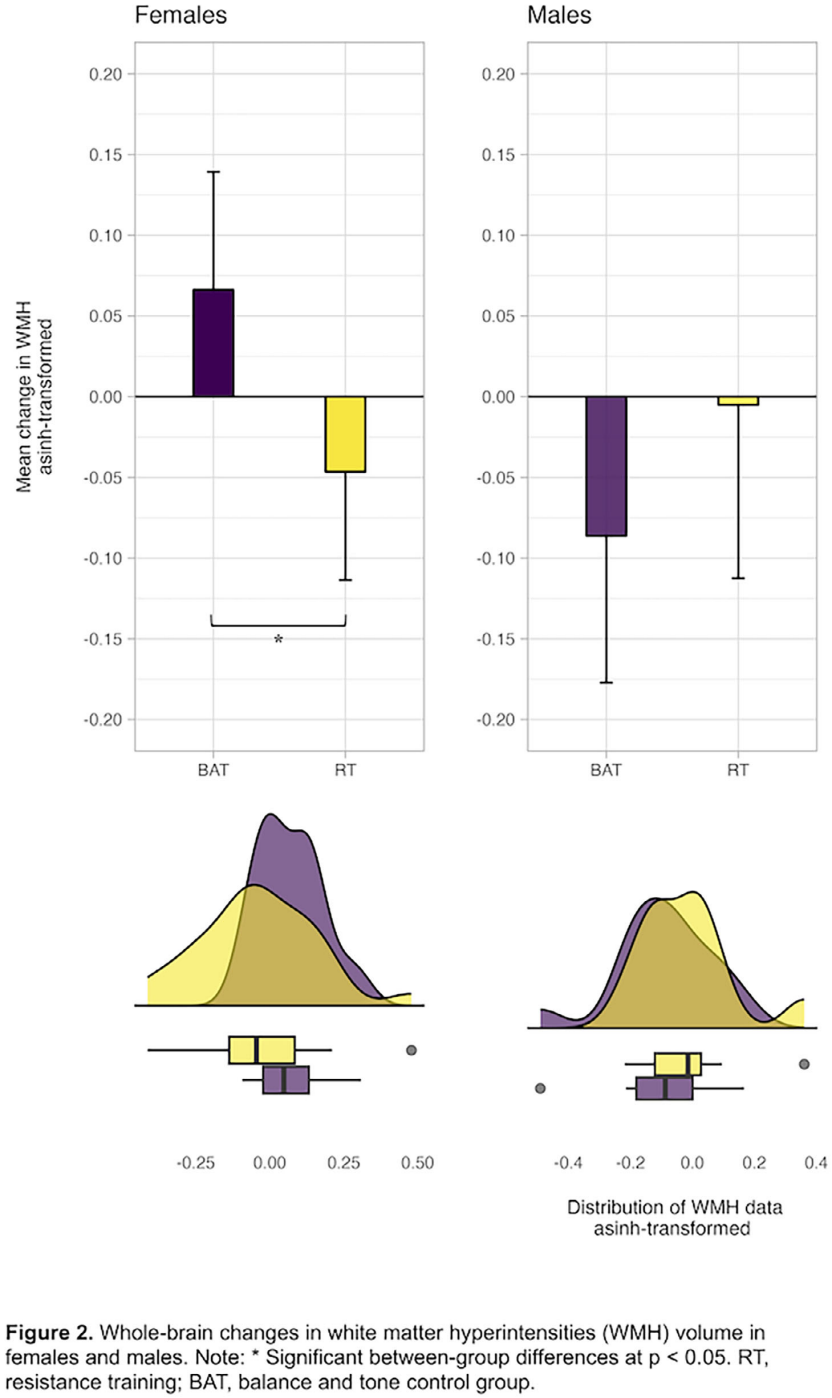# Exercise reduces white matter pathology in vascular cognitive impairment

**DOI:** 10.1002/alz.092848

**Published:** 2025-01-09

**Authors:** Narlon Cassio Boa Sorte Silva, Elizabeth Dao, Ryan S Falck, Walid Ahmed Alkeridy, Roger Tam, Kevin Lam, Cindy K Barha, Rachel A Crockett, Lisanne F ten Brinke, Thalia S Field, Teresa Liu‐Ambrose

**Affiliations:** ^1^ University of British Columbia, Vancouver, BC Canada; ^2^ Djavad Mowafaghian Centre for Brain Health, Vancouver, BC Canada; ^3^ Centre for Aging SMART, Vancouver Coastal Health Research Institute, Vancouver, BC Canada; ^4^ International Collaboration on Repair Discoveries, Vancouver, BC Canada; ^5^ King Saud University, Riaydh Saudi Arabia; ^6^ Libin Cardiovascular Institute, Calgary, AB Canada; ^7^ Hotchkiss Brain Institute, Calgary, AB Canada; ^8^ University of Calgary, Calgary, AB Canada; ^9^ University of Oxford, Oxford United Kingdom

## Abstract

**Background:**

Subcortical ischemic vascular cognitive impairment (SIVCI) is highly prevalent in older individuals. White matter hyperintensities (WMH) are a defining feature of SIVCI. There are sex differences in SIVCI, with females having higher volume and faster WMH progression than males. Resistance exercise training (RT) can slow WMH progression in cognitively unimpaired individuals; however, evidence in SIVCI is scarce. It is also unknown if biological sex influences WMH response to exercise. We investigated whether RT mitigated WMH in older individuals living with SIVCI and assessed whether biological sex moderated intervention effects.

**Methods:**

This was a 12‐month single‐blind, randomized controlled trial. Participants were randomized to RT (n = 45, females = 32) or a balance and tone (BAT) control group (n = 46, females = 29). Study eligibility included: 1) age 55 years and older; 2) magnetic resonance imaging (MRI) evidence of cerebral small vessel disease; 3) mild cognitive impairment; and 4) absence of dementia. We measured WMH using a seed‐based pipeline incorporating T2‐ and PD‐weighted MRI scans. We extracted volumes for whole‐brain and lobe‐specific regions‐of‐interest. Differences between groups from baseline to 12 months were assessed via analysis of covariance adjusting for baseline WMH volume, estimated intracranial volume, and sex. We assessed sex‐specific effects via group‐by‐sex interaction terms.

**Results:**

Seventy‐four participants (aged 74.3 [SD = 5.6], 65% females) completed follow‐up assessment and were included in the analysis. At 12 months, no main effect of intervention was observed for whole‐brain WMH volume (estimated mean difference [asinh‐transformed cm^3^]: ‐0.045, 95% CI: ‐0.124 to 0.034, p = 0.264). Biological sex significantly moderated intervention effects on whole‐brain WMH volume (p = 0.019), whereby RT reduced WMH volume (vs BAT controls) in females (‐0.113, 95% CI: ‐0.208 to ‐0.018, p = 0.021) but not males (0.081, 95% CI: ‐0.048 to 0.211, p = 0.216). Lobe‐specific analysis revealed that RT reduced WMH volume (vs BAT controls) in the parietal (‐0.074, 95% CI: ‐0.148 to ‐0.0004, p = 0.049) and temporal (‐0.069, 95%CI: ‐0.138 to ‐0.00003, p = 0.049) lobes. Group‐by‐sex interaction effects approached significance for parietal WMH volume (p = 0.052), suggesting an overall sex‐dependent effect of training.

**Conclusion:**

RT may be an effective strategy to mitigate WMH progression in older individuals with SIVCI and females likely reap greater benefits than males.